# Supplementary material for: The antiangiogenic peptide VIAN-c4551 inhibits lung melanoma metastasis in mice by reducing pulmonary vascular permeability
Source: PLoS One. 2025 May 28;20(5):e0316983. doi: 10.1371/journal.pone.0316983 (PMC12118997; doi:10.1371/journal.pone.0316983)
Supplement: Attachment — Submitted filename: Plos one report.docx [file pone.0316983.s001.docx]

The study titled **“The antiangiogenic peptide VIAN-c4551 inhibits lung melanoma metastasis in mice by reducing pulmonary vascular permeability”** demonstrates that **VIAN-c4551** effectively inhibits lung melanoma metastasis in mice by reducing pulmonary vascular permeability and preventing melanoma cell extravasation. The findings are well-supported by both in vivo and in vitro experiments, and the study opens new avenues for the development of anti-metastatic therapies. However, further research is needed to validate these findings in human clinical trials and to explore the full range of mechanisms by which VIAN-c4551 exerts its anti-metastatic effects.

To further evaluate the study’s findings and address potential limitations, the following questions could be posed to the authors:

**Dose Selection and Optimization**:

- Why was a single dose of VIAN-c4551 (1 mg/kg) chosen for the experiments? Have you conducted dose-response studies to determine the optimal therapeutic dose?
- Have you explored the pharmacokinetics of VIAN-c4551, such as its half-life, bioavailability, or potential accumulation in tissues, which could influence its efficacy and safety?

**Toxicity and Safety**:

- Did you evaluate the potential toxicity of **VIAN-c4551** in mice, particularly with long-term administration? Were there any signs of systemic toxicity, such as organ damage or immune suppression?
- Why did you not assess other serum markers (e.g., liver enzymes, kidney function) to evaluate the systemic impact of **VIAN-c4551** treatment?

**In Vitro Model Selection**:

- Why did you choose bovine umbilical vein endothelial cells (BUVEC-E6E7) for the in vitro experiments? Did you consider using human endothelial cell lines to better reflect human physiology?
- Why did you focus on venous endothelial cells rather than arterial endothelial cells, which might be more relevant to the pulmonary vasculature and metastatic seeding?
